# Outcome of Primary Hyperparathyroidism: Retrospective Tertiary Center Experience From Basrah, Iraq

**DOI:** 10.7759/cureus.65901

**Published:** 2024-07-31

**Authors:** Suha M Abdul Khaleq, Hussein A Nwayyir, Abbas A Mansour

**Affiliations:** 1 Diabetes and Endocrinology, Faiha Specialized Diabetes, Endocrine and Metabolism Center (FDEMC) University of Basrah, Basrah, IRQ

**Keywords:** cure rate, hyperparathyroidism, parathyroid surgery, hypercalcemia, parathyroidectomy, primary hyperparathyroidism

## Abstract

Background: Primary hyperparathyroidism is regarded as a common endocrine disorder that is biochemically identified and could be symptomatic or asymptomatic. A detailed history and a thorough evaluation with regular follow-ups are required until a definite diagnosis is made. The study aims to evaluate the characteristics of patients and the performance of a tertiary endocrine center in managing the disease in Basrah, Iraq.

Material and methods: A retrospective study was conducted at the Faiha Specialized Diabetes, Endocrine, and Metabolism Center in Basrah, southern Iraq, on 106 patients diagnosed with primary hyperparathyroidism between 2012 and 2023. The patients' general characteristics were assessed, and those who underwent parathyroidectomy were evaluated post-surgery, and the cure rate was determined.

Results: The mean age of presentation was 47.5 ± 14.6 years, with a median of 50 years. The highest occurrence is in the sixth decade. Females comprised 79 (75%) of the patients, and the female-to-male ratio was 3:1. Symptomatic patients were 84 (90%), 30 (70%) of the patients had nephrolithiasis, and 52 (68%) had osteoporosis. The cure rate was 15 (83%).

Conclusion: In our single-center study, the frequency of primary hyperparathyroidism has increased with time. The disease's highest occurrence was seen in the sixth decade. Females were substantially higher than males. Most patients were symptomatic. The cure rate was 83%.

## Introduction

Primary hyperparathyroidism (PHPT), a common endocrine condition characterized by hypercalcemia and high or inappropriately normal parathyroid hormone, is identified by biochemical tests [1]. There is growing recognition of normocalcemic PHPT as a less severe disease variant. The real incidence rate and frequency may be underestimated in this setting due to the underrecognition of milder forms of the condition [2].

Many epidemiological studies relied on patient referrals to secondary or tertiary health services. The reported prevalence of PHPT varies between 0.2% and 1.3% of the population worldwide, including the United States, Europe, Bahrain, and Korea [3]. The number of cases rises with age and despite that the disease can occur at any age, half of PHPT patients are postmenopausal women [1]. PHPT can manifest as symptomatic or without symptoms. Symptomatic individuals develop stones in the kidneys and osteoporotic fractures [4].

All patients should have their 25-hydroxy vitamin D levels measured since there is evidence that the disorder is more active in those who are vitamin D deficient or insufficient [5].

Parathyroid adenoma is the most common cause of primary hyperparathyroidism, whereas parathyroid carcinoma is rare and has an unfavorable outcome due to recurrence and metastasis. PHPT is caused by parathyroid hyperplasia in 10% of cases [6].

The only definite therapy for PHPT is parathyroid surgery [7]. Before surgery, localization methods assist in guiding the surgical strategy in patients with a biochemically proven disease diagnosis and in whom other diagnoses have been adequately excluded [8]. Neck ultrasonography (US), single photon emission computed tomography (SPECT), Tc-99m sestamibi imaging, magnetic resonance imaging (MRI), four-dimensional CT scan (4-D CT), and positron emission tomography in conjunction with CT scan are examples of non-invasive techniques that are employed [9]. Surgery should be done by a skilled parathyroid surgeon and is recommended based on several factors and results, including symptoms, age, skeletal involvement, renal involvement, and calcium level according to the guidelines from the Fifth International Workshop on the Evaluation and Management of Primary Hyperparathyroidism [10]. Bilateral neck exploration (BNE) and minimally invasive parathyroidectomy (MIP) are the two most popular surgical techniques for treating PHPT [11].

Individuals with PHPT who don't fit the criteria for parathyroidectomy can continue with regular follow-up. Medical options as recommended by the Panel are available for those who choose not to have surgery but meet specific guidelines, cinacalcet, calcium, vitamin D supplementation, alendronate, or denosumab are all used [10].

The present study aims to evaluate patients’ characteristics and the performance of a tertiary care endocrine center in Basrah, Iraq, in the management of primary hyperparathyroidism. 

## Materials and methods

Study design

**Figure 1:**
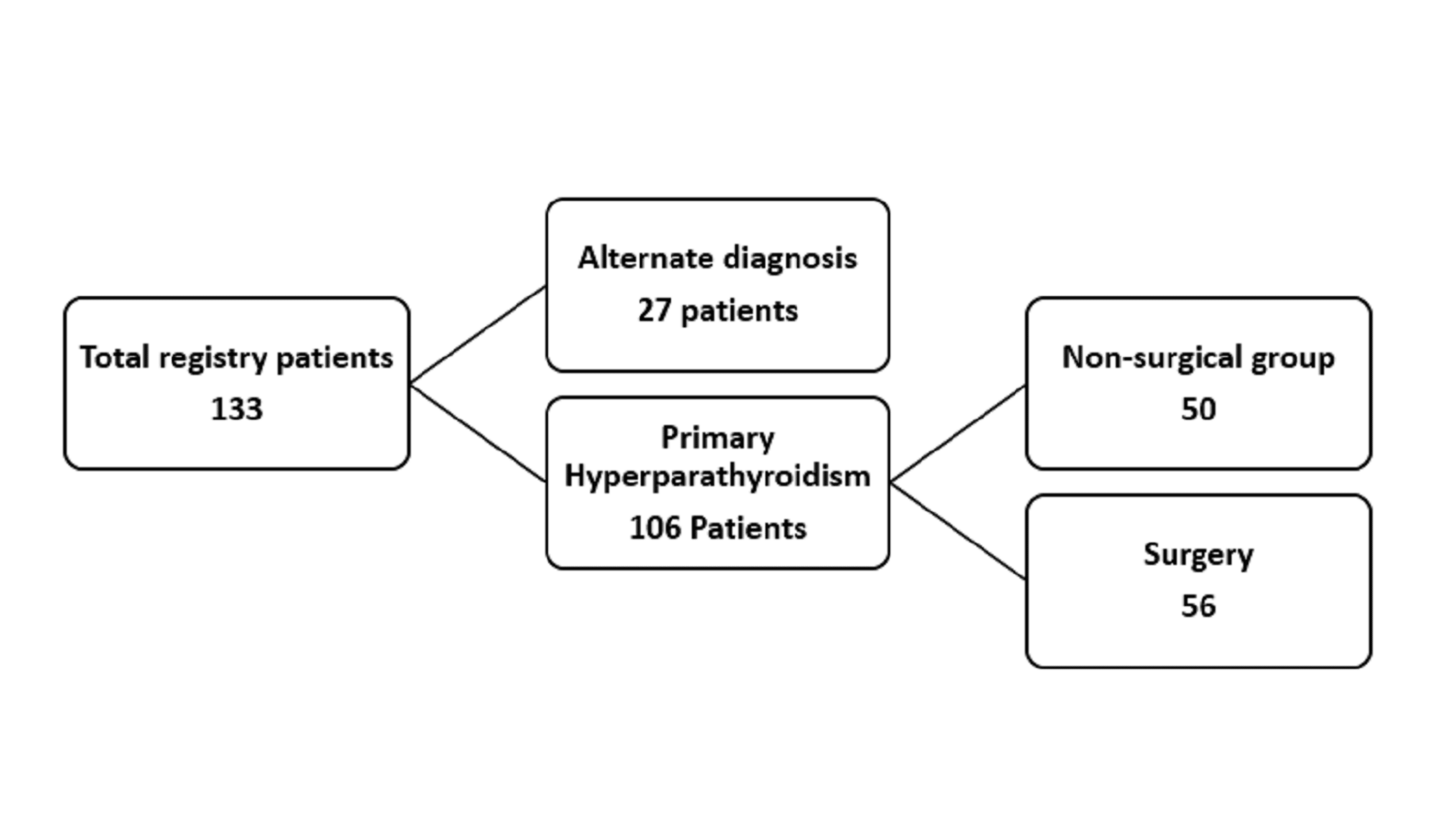
Flow chart of the total registry of primary hyperparathyroidism in Faiha Specialized Diabetes, Endocrine, and Metabolism Center (FDEMC)

This single-center retrospective study was conducted at the Faiha Specialized Diabetes, Endocrine, and Metabolism Center (FDEMC) in Basrah, southern Iraq in December 2023. Patients who met the criteria for the diagnosis of primary hyperparathyroidism [10] from 2012-2023 were included in the study. The FDEMC has an electronic database of medical records, and we used the keyword primary hyperparathyroidism to search for the registered data, and the cases were collected. As shown in Figure 1, there were 133 patients in the data registry; 27 of them appeared to be alternative diagnoses with subsequent follow-ups; the remaining 106 patients met the criteria for the diagnosis; 50 patients were in the non-surgical group who either refused surgery, stopped receiving follow-up care, continued receiving medication, or weren't candidates for surgery; and the other 56 patients had parathyroidectomy. Regarding localization, all 56 patients who underwent surgery had neck ultrasound and a 4-D CT. 

The inclusion criteria were patients who were 15 to 85 years old and patients who had met the diagnostic criteria for primary hyperparathyroidism [10]. Exclusion criteria were those who had a history of drugs causing hypercalcemia, a history of granulomatous diseases such as sarcoidosis or tuberculosis (TB), pregnant women, patients with chronic kidney disease, and patients whose age was below 15 years and above 85 years.

Hormonal and biochemical evaluation

Laboratory investigations were done in the form of serum calcium adjusted for albumin according to the formula "Corrected Calcium = Serum_calcium + 0.02 * (Normal_albumin - Patient_albumin)", serum parathyroid hormone (PTH), serum phosphorus, serum alkaline phosphatase according to the reference ranges of Roche (Basel, Switzerland) diagnostic kit [Total calcium: 8.2-10.2 mg/dL, albumin: 3.5-5.0 g/dL, intact PTH: 15-65 pg/mL, phosphate: 2.3 to 4.7 mg/dL, alkaline phosphatase: 40-150 U/L and 25(OH) D level cut off values were used (deficiency < 20 ng/ml, insufficiency 20-30 ng/ml and sufficiency > 30 ng/ml)]. The estimated glomerular filtration rate (eGFR) was also calculated by the Chronic Kidney Disease Epidemiology Collaboration (CKD-EPI) equation, and other hormonal biochemistry if needed was also measured. Twenty-four-hour urinary calcium has been used to help diagnose PHPT and for surgical indications. However, it was not affordable for most of our patients. Urinary calcium excretion was used to identify hypercalciuria >250 mg/day in women and >300 mg/day in men [10]. 

The fully automated Cobas e 411 electrochemiluminescence immunoassay was used for hormonal assays, and the Cobas c 311 analyzer system was used for clinical chemistry by Roche Diagnostics. Serum calcium, PTH, phosphorus, alkaline phosphatase, and vitamin D levels were measured pre-surgery and on first day, first week, first, third, and sixth month post-surgery. The cure rate was calculated according to those time frames. A surgical cure is defined as a normal calcium level at six-month follow-up in patients with hypercalcemia PHPT and a normal level of parathyroid hormone at six-month follow-up for normocalcemic patients [12].

Statistical analysis

The Statistical Package for the Social Sciences (SPSS), version 26.0 (IBM Corp., Armonk, NY, USA), was used to analyze the data. The numerical values (N) and percentages (%) were used to summarize the categorical variables and their frequencies. The mean ± standard deviations (M ± SD) were used to summarize continuous variables. Microsoft Excel version 18.2405.1121.0 (Redmond, WA, USA) was used for ratio calculations, tables, and graphs analysis. 

## Results

**Figure 2:**
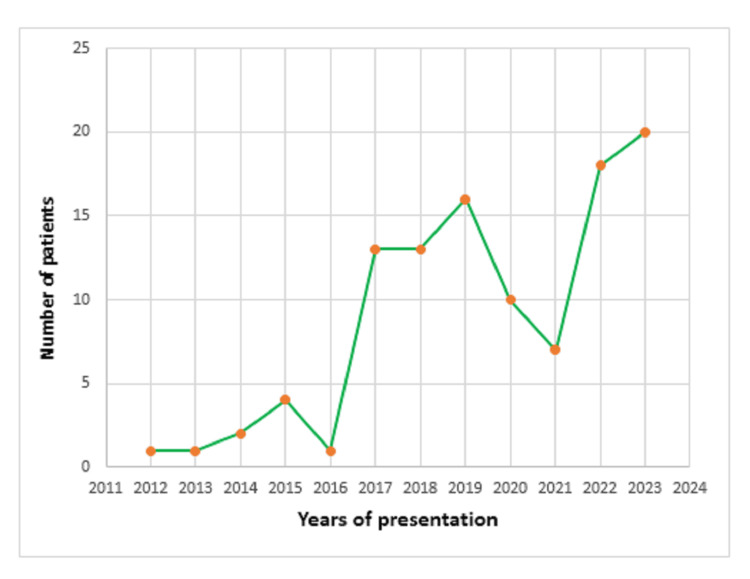
The diagnosis of the 106 patients with primary hyperparathyroidism (PHPT) at Faiha Specialized Diabetes, Endocrine, and Metabolism Center (FDEMC) over time.

The frequency of primary hyperparathyroidism in FDEMC has increased over the years, from one patient (0.9%) diagnosed in 2012 to 20 patients (19%) diagnosed by 2023, as shown below in Figure 2.

The demographics of the total number of patients with primary hyperparathyroidism are shown in Table 1. It was found that the mean age of presentation was 47.5 ± 14.6 years (the minimum age was 15 years and the maximum was 83 years), and the median was 50 years, with peak incidence in the sixth decade. The age mean ± SD in males was 44.1 ± 15.8 years, and the median was 45 years, while in females, it was 48.7 ± 14.1 years, and the median was 51 years.

**Table 1:** General characteristics of patients with primary hyperparathyroidism *number of patients with event/total number of patients analyzed (%) **Age mean ± SD in total patients

Variable	
Age (mean ± SD), years**	47.59 ± 14.6
Male (mean ± SD), years	44.1 ± 15.8
Female (mean ± SD), years	48.7 ± 14.1
Age (median), years	50
Female, n/N (%)*	79/106 (75%)
Symptomatic, n/N (%)*	84/93 (90%)
Nephrolithiasis, n/N (%)*	30/43 (70%)
Osteoporosis, n/N (%)*	52/76 (68%)
Fracture, n/N (%)*	22/104 (21%)
Brown tumor, n/N (%)*	16/106 (15%)
Vitamin D Deficiency, n/N (%)*	55/94 (58%)
Vitamin D Insufficiency, n/N (%)*	16/94 (17%)
Hungry bone syndrome, n/N (%)*	12/48(25%)

The females were 79 (75%) patients, which is more than the males, who were 27 (25%) patients, and the female:male ratio was 3:1. Menopausal women were 42/79 (53%), and non-menopausal women were 37/79 (47%). Of all patients, women with menopause were 42 (40%).

Ninety-three patients gave symptoms and histories; 84 (90%) of them were symptomatic, while the other nine (10%) were not. Nephrolithiasis was reported in 30 (70%) of patients who had renal ultrasonography. It was found that 52 (68%) patients out of 76 who had DEXA scans had osteoporosis. Fracture history was reported in 22 (21% of patients). A brown tumor was seen by 16 (15%) of the presented patients. Hungry bone syndrome was seen in 12 (25%) out of 48 post-surgical patients.

As for vitamin D deficiency, insufficiency, and sufficiency, it has been seen in 55 (58%), 16 (17%), and 23 (25%), respectively, of the 94 patients whose blood investigations have shown so.

Calcium levels were adjusted according to serum albumin as means ± standard deviations, and results showed serum calcium 12.5 ± 1.7 mg/dL, PTH level 869.1 ± 787.9 pg/mL, 25(OH)D levels 21.2 ± 12.8 ng/mL, serum phosphate 2.27 ± 0.7 mg/dL, and serum alkaline phosphatase 469.7 ± 458.8 U/L as shown in Table 2, along with post-surgical biochemical levels with means and standard deviations on the first, seventh, 30th, 90th, and 180th days are all mentioned in Table 2 below. The cure rate was calculated according to the level of calcium for hypercalcemic primary hyperparathyroidism patients and the PTH level for normocalcemic patients in subsequent periods after surgery. On the first day, 26 patients out of 39 patients had normalized serum calcium; on the first week and the first month, 17 patients out of 19 had normalized serum calcium; on the third month, 11 patients out of 16 had normalized serum calcium; and on the sixth month, 15 patients out of 18 had normalized serum calcium. The cure rates on the first day, first week, first month, third month, and sixth month were 67%, 89%, 89%, 69%, and 83%, respectively. 

**Table 2:** Biochemical investigations before and after surgery of the 56 surgical group patients with cure rate Abbreviations: PTH: parathyroid hormone; ALP: Alkaline phosphatase; SD: Standard Deviation; No.: number *The parameters (serum calcium, PTH, 25(OH)D, phosphate and ALP) ± Standard Deviation ** Total number of patients who had surgery in the pre-surgery column and those who attended follow-ups in the post-surgery columns

Parameter*	Pre-Surgery	1^st^ day	1 week	1 month	3 months	6 months
Serum calcium mg/dL	12.5 ± 1.7	9.6 ± 1.6	8.2 ± 1.4	8.2 ± 1.6	9.3 ± 1.3	9.3 ± 1.0
Serum PTH, pg/mL	869.1 ± 787.9	78.6 ± 185.7	208.9 ± 413	157.2 ± 148	201 ± 332.8	161.9 ± 220.5
Serum 25(OH)D, ng/mL	21.2 ± 12.8	19.7 ± 10.6	22.4 ± 10.6		31.1 ± 18.4	27.8 ± 14.7
Serum phosphate mg/dL	2.27 ± 0.7	1.8 ± 0.6	2.6 ± 0.7	3.4 ± 1.1	3.1 ± 0.8	3.1 ± 0.7
Serum ALP, U/L	469.7 ± 458.8	441.9 ± 375.5	627.5 ± 441.7	302.1 ± 188.3	173.5 ± 74.0	120.7 ± 63.7
Cure rate %		67 %	89 %	89 %	69 %	83 %
No. of patients**	56	39	19	19	16	18

## Discussion

To our knowledge, this is the first study conducted in Iraq that examined the general characteristics of patients with primary hyperparathyroidism and surgery outcomes in a tertiary endocrine facility.

Though we don’t have enough data regarding the incidence and prevalence of PHPT in our country, but the current study found that the number of cases of primary hyperparathyroidism in our center has risen with time, from a minimum value in 2012 to a handful of cases by 2023. The surge began after 2017, which might be attributed to the increased referral of cases and to routine calcium testing, which was included in the biochemical testing panel, and has improved since the advent of auto-analyzers. This is in concordance with a multi-center retrospective study from Saudi Arabia by Al‑Saleh et al. in Jeddah, Al Ahsa, and Riyadh with a total of 205 confirmed PHPT cases, where it concluded that just 24 PHPT patients were previously gathered over 16 years, whereas 205 new cases were identified within three years between 2016 and 2018 [13]. A drop in the diagnosis of new cases was seen from 2020 to 2021, and the reason behind this was mostly the coronavirus disease 2019 (COVID-19) pandemic. 

The patients' median age and the mean were calculated with the sixth decade exhibiting the highest frequency. This is compatible with a retrospective study carried out at the Bahrain Defense Force Military Hospital over three years by Abdulla and Suwaifi [14] and another conducted by Younes et al. at Jordan University Hospital [15] both found that those 50 years of age and older had a considerably higher overall prevalence of PHPT. Similar findings were also observed in a retrospective analysis of 62 cases of primary hyperparathyroidism operated on in the university hospital department of otolaryngology in eastern Algeria by Zitouni, with the 40-60-year-old age group being the most affected [16]. The study carried out by Al‑Saleh et al. [13] and another retrospective single-center study in Istanbul, Turkey by Unlu et al. [17] led to distinct results with higher means of age. This can be clarified by the fact that different age ranges were employed.

As for the sex distribution analysis of the current study, females outnumbered males three times. This is concordant with the studies Younes et al. [15] and the multicenter Saudi Arabian study by Al‑Saleh et al. [13]. In our current study, nearly half of the female participants were in menopause. These results are compatible with what has been mentioned in a review by Walker and Silverberg [1] and the observation that the disease appears to manifest in women between the ages of 50 and 60 years as stated by Bilezikian and Silverberg [18].

It was noticed that a minority of the patients had no symptoms. This is similar to what was reported by a study from the Indian PHPT registry by Arya et al. in 2021 [19] but it is still low when compared to another study conducted in Riyadh Saudi Arabia at King Khalid University Hospital by Malabu and Founda in 2007 [20] where nearly 24% of patients had no complaints at the time of diagnosis, and another Turkish retrospective study in Farabi Hospital, Department of Surgery by Usta et al. in 2015 [21] where 28% of patients were asymptomatic. These findings could be because of the enhanced biochemical screening brought about by the implementation of routine automated calcium tests in practice and the evolving health consciousness of the surrounding community, where the latter studies were carried out.

About three-quarters of individuals who had renal ultrasonography were discovered to have nephrolithiasis. It was close to the findings from a retrospective study at Sher‑i‑Kashmir Institute of Medical Sciences of 78 patients by Misgar et al. in 2016, in which 64% of patients had kidney stones [22]. This result was greater than the one found by Al‑Saleh et al. where kidney stones were seen only in 15% [13]. This higher percentage of nephrolithiasis might potentially be linked to the small number of patients who had imaging at that time with a large percentage of those who had nephrolithiasis. 

Osteoporosis was seen in more than half of the cases in the current study. It is similar to what was noted by Zitouni, where it was reported in 57% of patients [16]. It is more than previous studies, such as the study by Prasarttong-Osoth et al. conducted in Thailand at the Department of Surgery, Siriraj Hospital from 1997 to 2007, where 33% of patients had osteoporosis [23], and the one carried out by Al‑Saleh et al. where it was the most prevalent skeletal symptom seen in 32% of patients [13]. Our findings might be multifactorial reasons, given that osteoporosis was one of the common reasons for patient referral, and more than half of the females were in menopause.

In nearly one-quarter of patients, a history of fractures was identified. Similar findings were identified by Prasarttong-Osoth et al. where pathological fractures were noted in 20% [23] and also by Zitouni, where they were observed in 31% [16]. Of the overall patients, 15% had brown tumors. It is less than that observed in Algeria, where it has been found in 55% of cases.

In our study, more than half of patients had vitamin D deficiency and nearly one-third had insufficient levels. These results are similar to the general population as seen in a retrospective study done in FDEMC in 2021 by Hussein et al. where vitamin D deficiency was seen in 62.5% and insufficiency in 11.3% of patients [24].

Twenty-five percent of patients had hungry bone syndrome post-surgery. It resembles the study results by Zitouni, where it was observed in 19% [16]. It was higher than the Indian study by Misgar et al. which reported it in 10% of patients [22], and the Turkish study by Usta et al. where hungry bone syndrome was detected in 12% of cases [21].

In skilled facilities, the surgical cure rate for PHPT is around 95-99% [7]. In our center, the cure rate was found to be 83% six months after surgery. It is close to that observed in Algeria [16]. Our result was lower than that seen in the studies in Jordan [15], Turkey [17], Saudi Arabia [20], and India [22] where the cure rates in these studies were approximately 96% or more, but it was more than a retrospective study in Cairo, Egypt at the National Cancer Institute, where the cure rate was close to 74% [25]. In general, lower success rates may be related to multi-gland disease, patients with multiple endocrine neoplasia, type 1 (MEN 1), technical difficulties during the operation, and parathyroid adenoma that was overlooked because of an ectopic location as mentioned by Uludag et al. 2023 [26].

The limitations of the present study were that it was a single-center study. Most referrals were made after symptoms appeared; there might have been a bias in favor of symptomatic individuals and an underreporting of asymptomatic people. Some patients lost follow-up and failed to appear when scheduled, and finally, complications were not well mentioned.

## Conclusions

In our tertiary center study, the incidence of primary hyperparathyroidism is rising with time. The sixth decade featured the highest incidence of presentation. The disease is three-quarters more common in females than in males. The majority of patients have presented following the onset of skeletal and renal symptoms. The surgery cure rate was 83%. We need future studies to assess the performance after 10 years.

## References

[1] Walker MD, Silverberg SJ (2018). Primary hyperparathyroidism. Nat Rev Endocrinol.

[2] Wermers RA (2023). Incidence of primary hyperparathyroidism in the current era: have we finally reached a steady state?. J Clin Endocrinol Metab.

[3] Soto-Pedre E, Newey PJ, Leese GP (2023). Stable incidence and increasing prevalence of primary hyperparathyroidism in a population-based study in Scotland. J Clin Endocrinol Metab.

[4] Zhu CY, Sturgeon C, Yeh MW (2020). Diagnosis and management of primary hyperparathyroidism. JAMA.

[5] Grey A, Lucas J, Horne A, Gamble G, Davidson JS, Reid IR (2005). Vitamin D repletion in patients with primary hyperparathyroidism and coexistent vitamin D insufficiency. J Clin Endocrinol Metab.

[6] Sun B, Guo B, Wu B, Kang J, Deng X, Zhang Z, Fan Y (2018). Characteristics, management, and outcome of primary hyperparathyroidism at a single clinical center from 2005 to 2016. Osteoporos Int.

[7] Wilhelm SM, Wang TS, Ruan DT (2016). The American Association of Endocrine Surgeons guidelines for definitive management of primary hyperparathyroidism. JAMA Surg.

[8] Yip L, Silverberg S, Fuleihan GH, Rosen CJ (2024). Preoperative localization for parathyroid surgery in patients with primary hyperparathyroidism. UpToDate.

[9] Kowalski GJ, Buła G, Żądło D, Gawrychowska A, Gawrychowski J (2020). Primary hyperparathyroidism. Endokrynol Pol.

[10] Bilezikian JP, Khan AA, Clarke BL, Mannstadt M, Potts JT, Brandi ML (2022). The Fifth International Workshop on the evaluation and management of primary hyperparathyroidism. J Bone Miner Res.

[11] Singh Ospina NM, Rodriguez-Gutierrez R, Maraka S (2016). Outcomes of parathyroidectomy in patients with primary hyperparathyroidism: a systematic review and meta-analysis. World J Surg.

[12] Montgomery KB, Gillis A, Ramonell KM, Fazendin JM, Lindeman B, Chen H (2023). Comparative utility of preoperative imaging in normocalcemic versus hypercalcemic primary hyperparathyroidism. Am J Surg.

[13] Al-Saleh Y, AlSohaim A, AlAmoudi R (2022). Primary hyperparathyroidism in Saudi Arabia revisited: a multi-centre observational study. BMC Endocr Disord.

[14] Abdulla J, Suwaifi YM (2021). Prevalence and incidence of primary hyperparathyroidism in Bahrain: a retrospective study from one medical center. Neuro Endocrinol Lett.

[15] Younes NA, Al-Trawneh IS, Albesoul NM, Hamdan BR, Sroujieh AS (2003). Clinical spectrum of primary hyperparathyroidism. Saudi Med J.

[16] Nouikes Zitouni S (2021). Monocentric experience of primary hyperparathyroidism surgery in Algeria. Surg Open Sci.

[17] Unlu MT, Aygun N, Akgun IE, Yetkin SG, Erol RS, Isgor A, Uludag M (2021). Parathyroidectomy results in primary hyperparathyroidism: analysis of the results from a single center. Sisli Etfal Hastan Tip Bul.

[18] Bilezikian JP, Silverberg SJ (2000). Clinical spectrum of primary hyperparathyroidism. Rev Endocr Metab Disord.

[19] Arya AK, Kumari P, Bhadada SK (2021). Progressive rise in the prevalence of asymptomatic primary hyperparathyroidism in India: data from PHPT registry. J Bone Miner Metab.

[20] Malabu UH, Founda MA (2007). Primary hyperparathyroidism in Saudi Arabia: a review of 46 cases. Med J Malaysia.

[21] Usta A, Alhan E, Cinel A, Türkyılmaz S, Erem C (2015). A 20-year study on 190 patients with primary hyperparathyroidism in a developing country: Turkey experience. Int Surg.

[22] Misgar RA, Dar PM, Masoodi SR, Ahmad M, Wani KA, Wani AI, Bashir MI (2016). Clinical and laboratory profile of primary hyperparathyroidism in Kashmir Valley: a single-center experience. Indian J Endocrinol Metab.

[23] Prasarttong-Osoth P, Wathanaoran P, Imruetaicharoenchoke W, Rojananin S (2012). Primary hyperparathyroidism: 11-year experience in a single institute in Thailand. Int J Endocrinol.

[24] Hussein IH, Mansour AA, Nwayyir HA (2021). Total vitamin D3 (25-hydroxyvitamin D) concentrations in Basrah, Iraq. Biomed Pharmacol J.

[25] Mahmoud HG (2017). Clinical presentation and outcome of surgical management of primary hyperparathyroidism: a single center-based case series in Egypt. J Head Neck Physicians Surg.

[26] Uludag M, Unlu MT, Kostek M, Caliskan O, Aygun N, Isgor A (2023). Persistent and recurrent primary hyperparathyroidism: etiological factors and pre-operative evaluation. Sisli Etfal Hastan Tip Bul.

